# Sympathetic context of the disease - a new era in glaucoma management

**DOI:** 10.22336/rjo.2021.4

**Published:** 2021

**Authors:** Vasile Potop, Valeria Coviltir, Speranţa Schmitzer, Cătălina Ioana Ionescu, Miruna Gabriela Burcel, Maria Corbu, Dana Margareta Cornelia Dăscălescu

**Affiliations:** * Ophthalmology Department, “Carol Davila” University of Medicine and Pharmacy, Bucharest, Romania; ** Ophthalmology Department, Clinical Hospital of Ophthalmologic Emergencies, Bucharest, Romania; *** Ophthalmology Department, Oftaclinic Bucharest, Romania

**Keywords:** glaucoma, IOP, contextual IOP, etiology

## Abstract

Primary open angle glaucoma (POAG) is a multifactorial optic neuropathy, which progresses in a chronic manner. Several etiological factors are involved, including genetic factors, race, age, IOP or vascular, systemic factors. IOP has an established role in the initiation and evolution of glaucoma, but its interactions with additional risk factors are complex. We propose the notion of the Glaucoma Etiological Area (GEA), as a representation of all the elements acting in collaboration in the physiopathology of each glaucoma case. When combined in different proportions, these elements may trigger the typical glaucomatous optic neuropathy (GON). We know that the statistical values of IOP are valid for normal eyes, but the glaucoma eye is not a normal eye. The notion of GEA can open a new perspective to interpret IOP values and to assess the true value of IOP control as a treatment for glaucoma. Applying the GEA theory allows us to tune the role of IOP. Additional factors, such as ocular properties (RGCL status, CCT, IOP fluctuation curve), ocular comorbidities (PEX, PDS), systemic comorbidities (arterial hypertension, vasospastic diseases such as migraines or Reynaud’s syndrome) or patient’s attitude towards glaucoma management (treatment compliance, access to follow-up and treatment) may greatly influence the evolution of GON and should be viewed holistically when developing a management plan for each patient. Applying the notion of GEA in clinical practice allows a more realistic approach of the pathophysiology of the disease and for a glaucoma treatment that is tailored to each patient.

**Abbreviations:** AG = advanced glaucoma, BP = blood pressure, CCT = central corneal thickness, CIGTS = Collaborative Initial Glaucoma Treatment Study, CNTGS = Collaborative Normal-Tension Glaucoma Study, EMGT = Early Manifest Glaucoma Trial, GEA = glaucoma etiological area, GON = glaucomatous optic neuropathy, IOP = intraocular pressure, NTG = Normal Tension Glaucoma, OHTS = Ocular Hypertension Study, PDS = Pigmentary dispersion syndrome, PEX = Pseudoexfoliation syndrome, POAG - primary open-angle glaucoma, RGCL = retinal ganglion cell layer, VFL = visual field loss

## Introduction

POAG is a chronic, progressive, and multifactorial optic neuropathy. The multifactorial nature of the disease involves several etiological factors, from genetic factors, race, age to intraocular pressure and vascular factors [**1**]. 

The progression of glaucoma is a complex relationship between various etiological factors and retinal ganglion cell vulnerability [**2**,**3**]. This vulnerability increases with disease progression, which makes the glaucoma we are treating to be different over time, even if the eye is the same, as if the affected structure would become an accomplice to the disease [**2**,**3**].

The role of IOP in the appearance and progression of glaucoma has been proven. Glaucoma is generally perceived as a disease with a complex etiology, on which several risk factors act and surpass a certain threshold [**2**].

## The definition of the etiological area in glaucoma

As a condition with multiple risk factors, acting in different proportions, one might visualize glaucoma as an area, on which each etiological factor is proportionally represented. IOP may occupy a variable surface, itself having large fluctuations. 

The glaucoma etiological area (GEA) defines a critical value of the sum of etiologic factors that, once overcome, may trigger a glaucoma neuropathy.

Sometimes, the IOP is the only etiological factor that completes the etiological area to a critical value beyond which glaucoma occurs (e.g., exclusive hypertensive glaucoma). The progression of glaucoma is a complex relationship between various etiological factors and retinal ganglion cell (RGCL) vulnerability.

The notion of GEA allows a clearer understanding for the highly variable progression of glaucoma: as RGCL vulnerability increases with disease progression, the impact of previously recognized risk factors is changing [**4**-**6**]. Current treatments target IOP reduction, thus decreasing the overall GEA. Furthermore, the severity of glaucoma neuropathy is a vulnerability captured in the GEA. Therefore, treating glaucoma as early as possible may reduce the GEA, through control over glaucomatous neuropathy. 

Additionally, other risk factors comprising the GEA act alongside IOP and include modifiable (treatment compliance, systemic vascular factors, such as arterial tension) and non-modifiable risk factors (genetic factors, ocular comorbidities such as PEX or myopia). A patient’s GEA may be monofactorial (dominated by IOP values and fluctuation) or multifactorial/ plurifactorial (composed of several risk factors), in which case it may still be of limited diversity (IOP dominant) or truly diverse (several risk factors of comparable influence).

Including all such factors in the GEA allows for a clearer understanding of glaucoma initiation and evolution and a realistic IOP target reduction.

## IOP values and fluctuation in glaucoma progression

The current therapeutic paradigm of glaucoma revolves around IOP: a decrease in IOP leads to a delay in disease progression. The IOP-progression relationship is not linear and may change over the course of the disease. There is asymmetry of IOP values without asymmetry of progression [**7**]. *IOP acts as a surrogate parameter of the disease, and does not have a linear relationship with the disease. However, IOP control can prevent the progression of the disease in a large proportion of cases [**8**]*. 

Beyond the numeric value of IOP, glaucoma management may benefit from considering the evolution of IOP - the nictemeral pressure curve. IOP varies according to a nictemeral profile and the role of fluctuations is documented in literature [**9**,**10**]. The pressure curves may be influenced by other ocular elements of the GEA, such as PEX, PDS or angle closure. 

Several studies have focused on the complex relationship between IOP and glaucoma progression. If we convert the therapeutic results from NTG, OHTS, EMGGT into results that each doctor would achieve on an individual scale, they would be catastrophic. In these studies, the results have statistical value, in current practice they do not express a therapeutic success.

We need to ask ourselves why in OHTS 9.5% of the cases evolved to glaucoma without treatment? And of those with treatment, why 4.4% went to glaucoma despite treatment? A rate of therapeutic failure of about 50%. Through these results, OHTS has revealed both the role of TIO in the etiology of the disease and the role of other etiological factors.

*The OHTS study demonstrated the effects of IOP reduction in glaucoma [**11**]. The patients had a mean horizontal cup-disc ratio of 0.36, qualifying Humphrey 30-2 visual fields were normal and reliable, and baseline IOP varied between 24 and 32 mmHg. On average, the IOP was lowered by 22.5% in the study group and by 4.0% in the control, placebo group. After 5 years, the risk of developing POAG was 4.4% in the study group and 9.5% in the control group [**12**]*.

Risk factors for the development of POAG include age, cup-disc ratio, pattern standard deviation, IOP, but also central corneal thickness (CCT) - patients with a CCT of under 555 microns had a hazard ratio of 3.4 to developed OPAG, compared to those with a CCT of over 588 microns [**13**]. Thus, the OHTS established CCT as a relevant element of the GEA. Furthermore, hypotensive medication lowered the risk of developing POAG on long term - after 13 years, the proportion of glaucoma diagnosis was 16% in the study group and 22% in the control group [**14**].

CCT interacts with glaucoma in several ways - firstly, it may lead to under or overestimation of measured IOP. Secondly, a low CCT acts as an independent risk factor in glaucoma. Furthermore, CCT should be considered when establishing an IOP treatment goal [**15**].

Additionally, the moment in which the decision to treat is made is also of utmost importance: the EMGT study showed that, in patients diagnosed with early glaucoma, the decision to treat led to delayed and infrequent disease progression. 45% of the patients in the treatment group (laser trabeculoplasty plus topical betaxolol) progressed, compared to 62% of controls [**16**]. Factors that contribute to glaucoma progression are age, higher IOP (initially and after treatment initiation, at follow up visits), PEX, cardiovascular comorbidities, low ocular perfusion pressure and low systolic BP [**17**].

In the CIGTS study [**18**], newly diagnosed POAG was treated with either trabeculectomy or with a medical regimen, in a stepped manner. After 5 years, both groups have reported a similar evolution of VFL, and similarly lowered IOP (average of 17-18 mmHg in the medical group and 14-15 mmHg in the surgical group) [**19**]. Long-term results of the CIGTS study support the practice of adding medication in the therapeutic regimen, following IOP elevations of fluctuations, as these significantly raise the risk of VFL [**20**].

The CNTGS study followed NTG patients, and randomized them to either placebo or hypotensive treatment, with a 30% IOP reduction target [**21**]. 35% of the control eyes and 12% of the treated eyes showed VFL or cup/ disc ratio progression, the difference being statistically significant [**22**].

OHTS, EMGT, CIGTS and CNTGS are landmark studies, which translate well in the clinical practice of glaucoma. However, the target IOP reduction should be tailored to each patient and according to their GON and VFL evolution showing the impact etiological area on management plan.

In order to adequately treat POAG, clinicians should fully understand the etiology of each case. Therefore, assessing the GEA is the basis in understanding the pathogenesis and the way IOP influences the glaucoma progression.

GEA reflects the complexity of risk factors involved in each case and highlights the treatable areas of glaucoma. There may be cases with restricted GEA, such as hypertensive glaucoma, or cases with an extended GEA, such as NTG or PEX glaucoma.

One factor that should be included in all cases in the GEA is the RGCL resistance, and its protection may prove an efficacious adjuvant treatment path [**23**]. However, RGCL neuroprotection [**24**,**25**] is difficult to document, as the RGCL resistance to IOP fluctuations varies with the IOP values, glaucoma stages and other factors involved in the physiopathology of the disease. 

The GEA may be composed of a small number of highly influential risk factors, which could be easily identifiable (e.g., PEX, PDS) or less so (NTG, high myopia-associated glaucoma). Additionally, these major risk factors can act through several mechanisms, which in turn shape the GEA (e.g., in PEX, fibrillary material can cause trabecular obstruction, but an increased vulnerability of the optic nerve head has also been proven) [**26**-**30**].

In cases dominated by several other risk factors (e.g., NTG), normal IOP values may be sufficient to reach the critical threshold and lead to GON, and low values of IOP may be needed in order to control it) [**31**-**34**]. Therefore, despite IOP values in the normal statistical range, glaucomatous progression warrants additional investigations and raises the suspicion of diversity in the GEA. The impact of IOP on the GEA may vary, either through IOP fluctuations or through fluctuations in infusion pressure in those with NTG [**35**]. Only the IOP’s pressure curve corroborated with that of systemic arterial pressure can highlight such fluctuations of etiologic area.

In cases in which IOP is predominant in the GEA, its relationship with GON progression may be quasilinear [**36**], and the decrease in IOP can provide good control of the disease (e.g., angle closure glaucoma). Such cases can be described as having a unifactorial GEA. Cases of progression under normal IOP (maintained with treatment) allude to additional factors in the GEA.

Sometimes, the degree of neuropathy may explain IOP tolerance. When glaucomatous optic neuropathy (GON) is advanced, we must consider that the ON vulnerability is increased. Additional factors implied in the GEA, besides IOP, may shift “normal IOP values” (i.e., values that ensure glaucoma non-progression) below the statistical reference points of the normal values [**37**-**39**]. Values considered statistically normal may be an error factor in treatment efficiency. 

## The research future in glaucoma

Defining the complex physiopathology of glaucoma as the GEA leads to several important points both in research and in clinical practice. Firstly, it is important to determine *the true importance of IOP values in the etiology of the disease*. The range of normal IOP is between 9 and 21 mmHg. However, these values may still be accompanied by progressive GON in many patients. 

Secondly, glaucoma should be placed in the context of systemic health, as it may influence the IOP and the evolution of glaucoma. Diseases such as migraines, vasospasms, carotid obstructions, and nocturnal hypotension may lead to a low ocular tolerance for IOP [**40**-**42**]. Therefore, in some cases, only small IOP values are tolerated without severe consequences. 

Additionally, in some cases, the clinician needs to decrease dramatically the presence of IOP on the GEA, in order to control the influence of other factors in the GEA on the progression of the disease. Such cases include AG, in which maintaining IOP under the threshold of 12 mmHg is needed in order to control the GON [**32**,**43**,**44**]. Other risk factors occupy the GEA, and the IOP, although statistically normal, in that given etiological context, would have extended the etiological area beyond the tolerance of this eye with AG.

In cases of progressive GON and low IOP, the latter is essentially removed from the GEA, and the GON progresses under low tension conditions. Such low IOP may be due to systemic vascular dysregulation, and it poses a significant risk in POAG [**45**]. Pathophysiological changes are noted in such cases, including diminished perfusion of optic nerve head and oxidative stress [**46**].

The concept of GEA proves to be a useful critical thinking tool, as addressing the etiology of glaucoma as a unique and linear parameter (IOP) usually limits the nuance of therapy. The GEA allows the investigation of other risk factors and encourages the exploration of the IOP/ neuropathy/ whole organism interaction. Such exploration proves especially important in cases of progressive GON and low IOP, in which a multifactorial GEA is strongly suspected.

It motivates us to further reduce the apparently satisfactory IOP, to compensate for other factors in the etiological area of the disease. Behind an advanced and rapid progression glaucoma there may be various etiological factors such as ocular, from high IOP/ IOP fluctuating to ocular factors (disease form, comorbidity, stage of disease) or systemic factors.

In most cases, the GEA is multifactorial, thus using IOP exclusively as a parameter in treatment may lead to several errors [**47**]: 

*1. Underdiagnosing NTG, in which GON progresses under IOP values in the normal range*;

*2. Avoiding lowering IOP under normal statistical limits, in cases in which GON progresses further under treatment*;

*3. Equating risk of glaucoma progression with higher IOP values*.

The context of glaucoma comprises several ocular and systemic conditions, and the sum of all relevant or hypothetical risk factors is the etiological area of the disease. The assessment of this context forces us to compensate IOP to offset the effects of other risk factors. The context of the disease can guide us on the type of treatment; medical, laser or surgical treatment and may be viewed holistically, considering:

*1. Disease profile - the severity of structural and functional profile of the disease*;

*2. Eye context of the disease: secondary glaucoma, associated pathologies*;

*3. Extra-ocular associated morbidities: i.e., diabetes, arterial hypertension, Parkinson’s disease*;

*4. Social condition of the patient: accessibility to treatment*.

## Conclusions

Analyzing IOP values and fluctuations in the complex context of the patient, following the step-up approach highlighted previously, has the potential to significantly improve glaucoma control and to control GON progression. The GEA encourages the clinician to carefully review these aspects and obtain the right IOP values, using the right therapeutic tools, for each glaucoma patient.

The GEA, or the etiological context of each glaucoma case, may be summed up as follows:

OpticNEuropathy, EYE SIMULTaneous and PATIent COmorbidities Contextus

ONE EYE SIMPATICO CONTEXTUS

ONE EYE SIMPATICO CONTEXTUS - A context that sympathizes with the disease

**Conflict of Interest**

None.

**Acknowledgements**

All authors have equal contribution to this paper.

**Sources of Funding**

None. 

**Disclosures**

None.
